# Health-economic evaluation of orthogeriatric co-management for patients with pelvic or vertebral fragility fractures

**DOI:** 10.1186/s12877-024-05225-5

**Published:** 2024-08-05

**Authors:** Espen Henken, Hans-Helmut König, Clemens Becker, Gisela Büchele, Thomas Friess, Andrea Jaensch, Kilian Rapp, Dietrich Rothenbacher, Claudia Konnopka

**Affiliations:** 1 Department of Health Economics and Health Services Research, University Medical Center Hamburg-Eppendorf, Martinistr. 52, 20246 Hamburg, Germany; 2 Department of Clinical Gerontology, Robert-Bosch-Hospital, Stuttgart, Germany; 3 Institute of Epidemiology and Medical Biometry, Ulm University, Ulm, Germany; 4 AUC - Akademie der Unfallchirurgie GmbH, Munich, Germany

**Keywords:** Orthogeriatric co-management, Insurance claims data, Health-economic evaluation, Pelvic fractures, Vertebral fractures

## Abstract

**Background:**

Orthogeriatric co-management (OGCM) addresses the special needs of geriatric fracture patients. Most of the research on OGCM focused on hip fractures while results concerning other severe fractures are rare. We conducted a health-economic evaluation of OGCM for pelvic and vertebral fractures.

**Methods:**

In this retrospective cohort study, we used German health and long-term care insurance claims data and included cases of geriatric patients aged 80 years or older treated in an OGCM (OGCM group) or a non-OGCM hospital (non-OGCM group) due to pelvic or vertebral fractures in 2014–2018. We analyzed life years gained, fracture-free life years gained, healthcare costs, and cost-effectiveness within 1 year. We applied entropy balancing, weighted gamma and two-part models. We calculated incremental cost-effectiveness ratios and cost-effectiveness acceptability curves.

**Results:**

We included 21,036 cases with pelvic (71.2% in the OGCM, 28.8% in the non-OGCM group) and 33,827 with vertebral fractures (72.8% OGCM, 27.2% non-OGCM group). 4.5–5.9% of the pelvic and 31.8–33.8% of the vertebral fracture cases were treated surgically. Total healthcare costs were significantly higher after treatment in OGCM compared to non-OGCM hospitals for both fracture cohorts. For both fracture cohorts, a 95% probability of cost-effectiveness was not exceeded for a willingness-to-pay of up to €150,000 per life year or €150,000 per fracture-free life year gained.

**Conclusion:**

We did not obtain distinct benefits of treatment in an OGCM hospital. Assigning cases to OGCM or non-OGCM group on hospital level might have underestimated the effect of OGCM as not all patients in the OGCM group have received OGCM.

**Supplementary Information:**

The online version contains supplementary material available at 10.1186/s12877-024-05225-5.

## Background

Fragility fractures are fractures caused by a low-trauma event, typically a fall from standing height or less [1]. Their incidence increases with age [2–4] and the lifetime risk for a fragility fracture from the age of 50 is estimated at 35% for women and 20% for men in Germany – estimates comparable to risks of stroke or cardiovascular disease [5]. Furthermore, a substantial economic burden is associated with fragility fractures [6, 7]. In the European Union (plus the United Kingdom and Switzerland) the annual costs were estimated at €56.9 billion for 2019 [8]. As in Germany [9] and many other countries the proportion of older persons is expected to increase, the health and economic burden of fragility fractures are expected to increase further [3, 5, 10].

### Burden of pelvic and vertebral fractures

The consequences of fragility fractures are closely related to their location [3, 11]. As hip fractures are associated with the severest health consequences [12] and highest healthcare expenditures [5] among all fragility fractures, they were the focus of most studies investigating fragility fractures [13]. However, since most of the fragility fractures are non-hip fractures [3, 5, 6], it is important to shed light on other fracture locations and their impact on healthcare systems. Two particularly burdensome non-hip fractures associated with increased mortality are pelvic [14–16] and vertebral fractures [17–19]. One study even showed that the mortality associated with pelvic ring fractures is similar to that of hip fractures [20]. In addition, a study showed a similar risk for institutionalization after pelvic, vertebral, and hip fractures [21]. While incidence rates for pelvic fractures are distinctly lower than for hip fractures [22, 23] their incidence is expected to increase in many European countries [22, 24–27]. Vertebral fractures, however, are among the most frequent or even *the* most frequent fragility fractures [5, 28–30].

Although pelvic fractures are associated with a substantial economic burden [31], they are often categorized as *other fractures* in studies on fragility fractures (e.g. in [6, 23, 28]). Hence, their share of healthcare expenditures is unclear. Vertebral fractures, however, are often argued to have the second highest economic burden after hip fractures [32, 33]. Thus, pelvic and vertebral fractures are severe fragility fracture locations that deserve more attention to mitigate their health and economic burden.

### Orthogeriatric co-management

Most geriatric patients with fragility fractures suffer from several comorbidities and eventually, frailty that require special care in addition to the treatment of the fracture. However, addressing comorbidities is often beyond the scope of surgical treatment [34]. Therefore, comprehensive care models have been developed [35–39].

Currently, different models of co-management of orthopedic surgeons and geriatricians exist in different countries. They range from an orthopedic treatment with on-demand consultation by a geriatrician to a jointly shared responsibility of orthopedic surgeons and geriatricians in a dedicated orthogeriatric ward [40]. In Germany, orthogeriatric co-management (OGCM) is often applied in such a way that allows reimbursement of the operations and procedures code (OPS) 8-550 – complex early geriatric rehabilitation [41]. This describes joint care of a geriatrician-led multidisciplinary team of geriatricians, orthopedic surgeons, physiotherapists, occupational therapists, specially trained nurses, and social workers either applied in an orthopedic or geriatric ward [34]. Treatment components are standardized geriatric assessment, regular interdisciplinary team meetings, and the development of a rehabilitation plan. The key element is an early mobilization [34].

Multiple studies, primarily on hip fractures, showed that co-management of orthopedic surgeons and geriatricians can improve health outcomes. Three systematic reviews summarized the evidence regarding orthogeriatric care for hip fractures and found benefits of orthogeriatric care such as a decreased in-hospital and 1-year mortality, higher osteoporosis treatment rates, or decreased healthcare costs after acute admission and 12–18 month follow-up [42–44]. The authors of both reviews with economic outcomes suggested these treatment models to be cost-effective [42, 43]. However, most of the included studies found a decreased length of stay for patients treated with orthogeriatric care while multiple investigations in Germany did not find a reduced [34, 45–48] or even found a longer length of stay [34, 45, 48]. This likely can be attributed to the reimbursement scheme of OPS 8-550, which requires a minimum stay of 14 days to qualify for a higher reimbursement. Accordingly, a German study found treatment in hospitals that offer OGCM only to be cost-effective at a willingness-to-pay of at least €82,000 per life year gained [45].

For pelvic or vertebral fractures, neither effectiveness nor cost-effectiveness has been widely investigated yet. A few studies compared short-term outcomes of patients with inter alia subtypes of pelvic or vertebral fractures treated in hospitals before or after the establishment of orthogeriatric care. They found benefits of orthogeriatric care concerning improved identification of complications [46, 49], fewer revision surgeries necessary [46], as well as higher rates of osteoporosis treatments and improved post-operative mobilization [46, 47]. However, one study on vertebral and other fractures found no differences concerning re-admission after 30 days or postoperative complications [50]. None of the studies found differences regarding mortality [46, 47, 49], although one showed a slight decrease in the OGCM group [48]. Results regarding length of stay were inconclusive – studies did not find a difference [46, 47, 50], showed a prolonged length of stay in OGCM [48], or a slight decrease [49]. Considering that many of these studies were conducted in Germany, it is important to highlight the high rate of vertebral fracture patients being treated operatively in Germany compared to the UK or other NHS countries. Overall, OGCM might be beneficial for the treatment of pelvic and vertebral fragility fractures, but to date, there is no health-economic evaluation.

### Research questions

This study aimed to analyze the costs and cost-effectiveness of the treatment of geriatric patients with pelvic or vertebral fragility fractures either in OGCM or non-OGCM hospitals observed for a 1-year follow-up period. The situation in Germany in the last decade reflects an optimal time window for such an investigation as an increasing number of hospitals were implementing OGCM. Therefore, hospitals that had not or not yet implemented OGCM could be compared with those that already had.

## Methods

### Data and study design

We used data from the *Allgemeine Ortskrankenkasse* (AOK), Germany’s largest association of health insurance companies that covers about one-third of the German population. The WIdO (*Wissenschaftliches Institut der AOK*), the scientific institute of the AOK, provided us with complete health and long-term care insurance claims data for the years 2013 to 2019. Health insurance is mandatory in Germany and most persons (about 90% of the population) are insured in statutory health insurances such as the AOK. Only self-employed persons or those with an income above a certain threshold can chose a private insurance (plus a few other groups). Although there are slight differences between insurance types, essential services are reimbursed by both and in inpatient setting, reimbursement mostly is the same for private and statutory insurances. We conducted a retrospective cohort study with continuously insured patients (insured for at least 90 days within a quarter and 360 days within a year). Of each patient, we considered hospital stays per fragility fracture location between 2014 and 2018 with either pelvic (ICD-10: S32.1, S32.3, S32.4, S32.5, S32.81, S32.83) or vertebral fractures (ICD-10: S12.0, S12.1, S12.2, S12.7, S.12.9, S22.0, S22.1, S32.0) as discharge diagnosis. Moreover, we included fracture cases with an inpatient hospital stay with the discharge code “M80” (i.e., osteoporosis with pathological fracture) and one of the above-mentioned ICD-10 codes as admission or secondary diagnosis. We excluded cases with multiple fragility fracture locations – pelvic, vertebral, humeral (S42), forearm (S52), or hip fractures (S72.0, S72.1) – as secondary diagnoses as these could not be assigned unambiguously to one of the fracture cohorts.

To identify OGCM from the claims data, the procedure code OPS8-550 can be used. However, this OPS code can only be used if a treatment lasted for at least 7 (8-550.0), 14 (8-550.1), or 21 days (8-550.2) with 14 days (8-550.1) triggering a higher reimbursement rate [51]. Consequently, using this OPS code for group assignment on case-level would introduce an immortal time bias [52] because this code can only be used when patients have survived for at least 7, 14, or 21 days. In line with similar studies [34, 45, 53, 54], we applied a hospital-level approach assigning cases to OGCM or non-OGCM group depending on whether the first treating hospital was able to offer OGCM at the day of admission. We used a categorization provided by the WIdO that defined OGCM hospitals if at least 10 OPS8-550 were reimbursed in a respective year. We also defined hospitals as OGCM hospitals when they had not reimbursed 10 of these OPS in one year but in prior and subsequent years, assuming that they were able to provide OGCM in the meantime. While this approach makes it difficult to relate differences to the actual application of OGCM, we assume that patients treated in an OGCM hospital might benefit from the existing multidisciplinary team even if OPS 8-550 was not applied.

We excluded cases of patients younger than 80 at the date of admission to ensure that all patients were geriatric [55]. Furthermore, we excluded cases treated in hospitals that often transferred patients to hospitals with a different OGCM status (i.e., non-OGCM hospitals transferring to OGCM hospitals and vice versa) to ensure that cases assigned to a group were actually treated in an OGCM hospital or non-OGCM hospital, respectively. For this, we calculated the proportion of pelvic and vertebral fractures in 2013–2019 with an OGCM status change for each hospital (considering only the first and last stay), calculating two proportions if a hospital itself changed OGCM status during this time. Then, we excluded all cases in hospitals with more than 5% status changes. In addition, we excluded cases that were treated in a hospital with a uniquely high number of fracture cases to allow an adequate risk adjustment. Moreover, we excluded a case with implausibly low index stay costs (€0.01). We only used the first valid hospital stay per person and fracture location. Then, we excluded cases of patients who were not insured for the entire baseline and follow-up period (except when they died during the latter) and excluded cases with a preceding fracture of the same location within 180 days before admission to focus on incident fractures. Lastly, we excluded patients with a hospital stay recorded after their day of death (see supplementary Fig. 1 for a flow-chart). Starting from the day of admission of the initial hospital stay, we applied a 1-year baseline and follow-up period.

### Outcomes

Regarding economic outcomes, we analyzed healthcare costs per sector, total healthcare costs as the sum of all sectors, and LOS. Regarding LOS, we also considered the length of all consecutive inpatient stays (stays with admission date on or before the discharge date of the index). Moreover, we added the length of the first inpatient rehabilitation stay in the 4 weeks after index hospital discharge to address that rehabilitation measures after fracture treatment often are part of an inpatient stay in OGCM but a provision in subacute rehabilitation facilities is more common in non-OGCM hospitals. We also report the in-hospital and rehabilitation facility length of stay separately. We investigated the following cost sectors: inpatient hospital treatments (including inpatient rehabilitation and index stay), index hospital stay (including costs for consecutive stays with the same fracture location and including associated inpatient rehabilitation), medications, outpatient treatments, outpatient hospital treatments, medical devices/medical appliances, and long-term care. We report all costs in 2019 Euro and adjusted them for inflation with the Gross Domestic Product price index [56]. To avoid bias by extreme outliers, we winsorized all costs at the 99% percentile. Based on health insurance data, this study took a payer perspective.

We could only obtain costs for long-term care indirectly: In Germany, long-term care recipients are categorized by care levels 1–5 which depend on the impairment of the ability to manage activities of daily living [57]. Information on care levels and care setting (home care or nursing home) was available per monthly period. The monthly reimbursement rate is fixed per care level depending on the care setting. Thus, we calculated long-term care costs by multiplying the months per care level with the respective reimbursement rate, which we obtained from the Federal Ministry of Health’s website [57]. In addition, the reimbursement rate for ambulatory care depends on whether it is delivered as benefits-in-kind by a professional care service or informally. As we did not know which of the two was reimbursed to which extent, we used the average of both rates. Lastly, if a person stays in a hospital for more than 28 days, no reimbursement for long-term care is paid that month. Thus, we subtracted the proportion of days in each month in which inpatient length of stay exceeded 28 days before summing the months within a respective care level.

As the primary effectiveness outcome, we estimated the survived time within the 1-year follow-up (*life years)*. Information on patients’ deaths was available only per monthly period. Therefore, we used the number of insured days within the last available quarter to approximate an exact date of death. As a secondary effectiveness measure, we used *fracture-free life years*, because many studies argued that the risk for a subsequent fracture of any location increases after an initial fragility fracture [58–60]. We calculated the time between index fracture and death or a subsequent fragility fracture of any location diagnosed in inpatient (defined by the discharge diagnosis) and outpatient settings (defined by the main diagnosis). For this, we also considered humeral (S42), forearm (S52), and hip fragility fractures (S72.0, S72.1). To distinguish re-fracture from re-treatment, we did not consider fractures of the same location as the index fracture within the first 6 weeks.

### Risk adjustment

As this was an observational study, we had to account for potential biases and unbalanced baseline characteristics caused by a lack of randomization. Thus, we applied *Entropy Balancing* (EB; [61]) which weights the individuals in the control group in such a way that the moments (i.e., means, variances, and skewness) of the covariates in the control group mirror those in the study group. If two groups are balanced on relevant covariates, group differences in the outcome can be better related to the grouping variable. Multiple studies demonstrated that EB achieves more balanced covariate distributions than other common approaches like propensity score weighting [61–63]. For EB, we used gender, age, and treatment year at admission. Moreover, we used 22 medication-based comorbidities [64], months in a nursing home, months with care level 1 to 5, and costs from all healthcare sectors during baseline. Lastly, we balanced for hospital volume. To obtain the hospital volume – the amount of pelvic or vertebral fracture cases within each hospital – we counted all pelvic and vertebral fracture cases from 2013 to 2019, respectively. To account for regional differences in the AOK’s insurant coverage, we weighted the hospital volume by the coverage in the patients’ federal states of residence. To obtain the coverage, we divided the number of AOK-insured persons per federal state [65] by the population of the respective federal state [66]. As limited overlap in the covariate distributions of the study and control group may impede adequate balancing, we excluded comorbidities with less than 50 observations (HIV, migraines, and tuberculosis) and cases treated in an OGCM hospital with a uniquely high hospital volume (supplementary Fig. 2). We used the weights of the EB for all statistical analyses.

### Statistical analysis

To account for typically right-skewed healthcare costs, we analyzed total, inpatient, and index stay costs with generalized linear models with a gamma distribution and a log-link [67]. In addition, we analyzed total and hospital length of stay with these models. We applied two-part models to analyze medication, outpatient, outpatient hospital, medical devices costs, and length of stay in rehabilitation facilities. For the first part, we used logistic regressions to estimate the probability of costs (or rehabilitation) occurring and for the second part, we used generalized linear models with a gamma distribution and a log-link to model the amount of costs (or length of stay in rehabilitation facility) for all non-zero values. We used *t*-tests to analyze long-term care costs, life years, and fracture-free life years.

We calculated the incremental cost-effectiveness ratio (ICER) as the ratio of the weighted mean difference of total costs and the weighted effectiveness difference between OGCM and non-OGCM groups for both effectiveness measures, respectively. Thus, the ICER describes either the costs per additional life year or the costs of an additional fracture-free life year due to treatment in OGCM hospitals compared to treatment in non-OGCM hospitals. Moreover, we applied net-monetary benefit regressions to estimate the probability that treatment in an OGCM hospital was cost-effective for different willingness-to-pay thresholds [68]. For this, we iterated the willingness-to-pay between €0 and €150,000 in steps of €1000 per iteration. We report the results in cost-effectiveness acceptability curves, CEACs [69], and considered treatment in an OGCM hospital cost-effective if its probability of being cost-effective exceeded 95%.

In a sensitivity analysis, we recalculated all analyses accounting for potential clusters introduced by cases from the same hospitals by using random intercept terms for hospitals. Moreover, balancing for hospital volume led to high weights for cases in a few large hospitals in the control group. To rule out that the results were driven by cases treated in these hospitals, we calculated sensitivity analyses without balancing for hospital volume. For a sample description, we calculated the proportion of surgical treatments among all cases as indicated by OPS 5-798 and OPS 5–79 with d as the sixth digit, e.g., 5-790.0d or 5-790.nd, for pelvic fractures and OPS 583 for vertebral fractures. For all analyses, we set α = 0.05 and used SAS software v9.4 (SAS Institute Inc, Cary, NC), R (version 4.2.0), and Stata 16 (StataCorp, College Station, TX). The funding source ensured the authors’ independence in designing the study, interpreting the data, writing, and publishing the report.

## Results

We included 21,036 cases with pelvic (71.2% in the OGCM, 28.8% in the non-OGCM group) and 33,827 with vertebral fractures (72.8% OGCM, 27.2% non-OGCM group). The baseline characteristics (before and after EB) can be found in Table 1 and supplementary Table 1 for pelvic and supplementary Table 2 for vertebral fractures. We obtained pre-balancing group differences in both cohorts regarding hospital volume and treatment years. After EB, the means and variances of both groups matched closely in both cohorts. The weighted mortality rate was 27.7% in the OGCM and 27.9% in the non-OGCM group for pelvic fracture patients. In the vertebral fracture cohort, the rates were 24.8% in the OGCM and 24% in the non-OGCM group. A surgical treatment was recorded for 5.9% (OGCM) and 4.5% (non-OGCM) of the pelvic and 33.8% (OGCM) and 31.8% (non-OGCM) of the vertebral fractures.

**Table 1:** Descriptive statistics before and after EB for pelvic fractures

Baseline: 1 year	OGCM group (*N* = 14,973)	Non-OGCM group (*N* = 6,063)	
		Before EB	After EB
Female gender [%]	84.55 (36.14)	84.64 (36.06)	84.55 (36.14)
Age: Mean [years]	87.37 (4.56)	87.26 (4.53)	87.37 (4.56)
Pelvic fracture cases per hospital: Mean	319 (162)	254 (134)	319 (162)
Treatment in 2014 [%]	14.47 (35.18)	22.02 (41.44)	14.47 (35.18)
Treatment in 2015 [%]	16.77 (37.36)	20.88 (40.65)	16.76 (37.35)
Treatment in 2016 [%]	20.54 (40.4)	19.38 (39.53)	20.53 (40.39)
Treatment in 2017 [%]	22.77 (41.94)	18.84 (39.1)	22.76 (41.93)
Care dependence during baseline: Mean [months]			
nursing home	1.84 (4.09)	1.79 (4.04)	1.84 (4.09)
care level 1	0.05 (0.62)	0.05 (0.6)	0.05 (0.62)
care level 2	3.13 (4.86)	2.9 (4.73)	3.13 (4.86)
care level 3	2.11 (4.19)	2.13 (4.21)	2.11 (4.19)
care level 4	1.16 (3.28)	1.11 (3.19)	1.16 (3.28)
care level 5	0.15 (1.19)	0.16 (1.25)	0.15 (1.19)
Costs during baseline: Mean [€]			
for inpatient hospital treatment	4,219 (6,969)	4,000 (6,808)	4,220 (6,969)
for medication	1,279 (1,520)	1,251 (1,477)	1,279 (1,520)
for outpatient treatment	1,340 (2,858)	1,377 (2,851)	1,340 (2,858)
for outpatient hospital treatment	20.48 (99.31)	20.99 (99.58)	20.46 (99.27)
for medical devices	168 (394)	185 (420)	168 (394)
for long-term care	6,142 (6,498)	6,030 (6,586)	6,142 (6,498)

Standard deviation is stated in parentheses; EB = Entropy balancing; OGCM = Orthogeriatric co-management

Medication-based comorbidities are depicted in supplementary Table 1

**Fig. 1:**
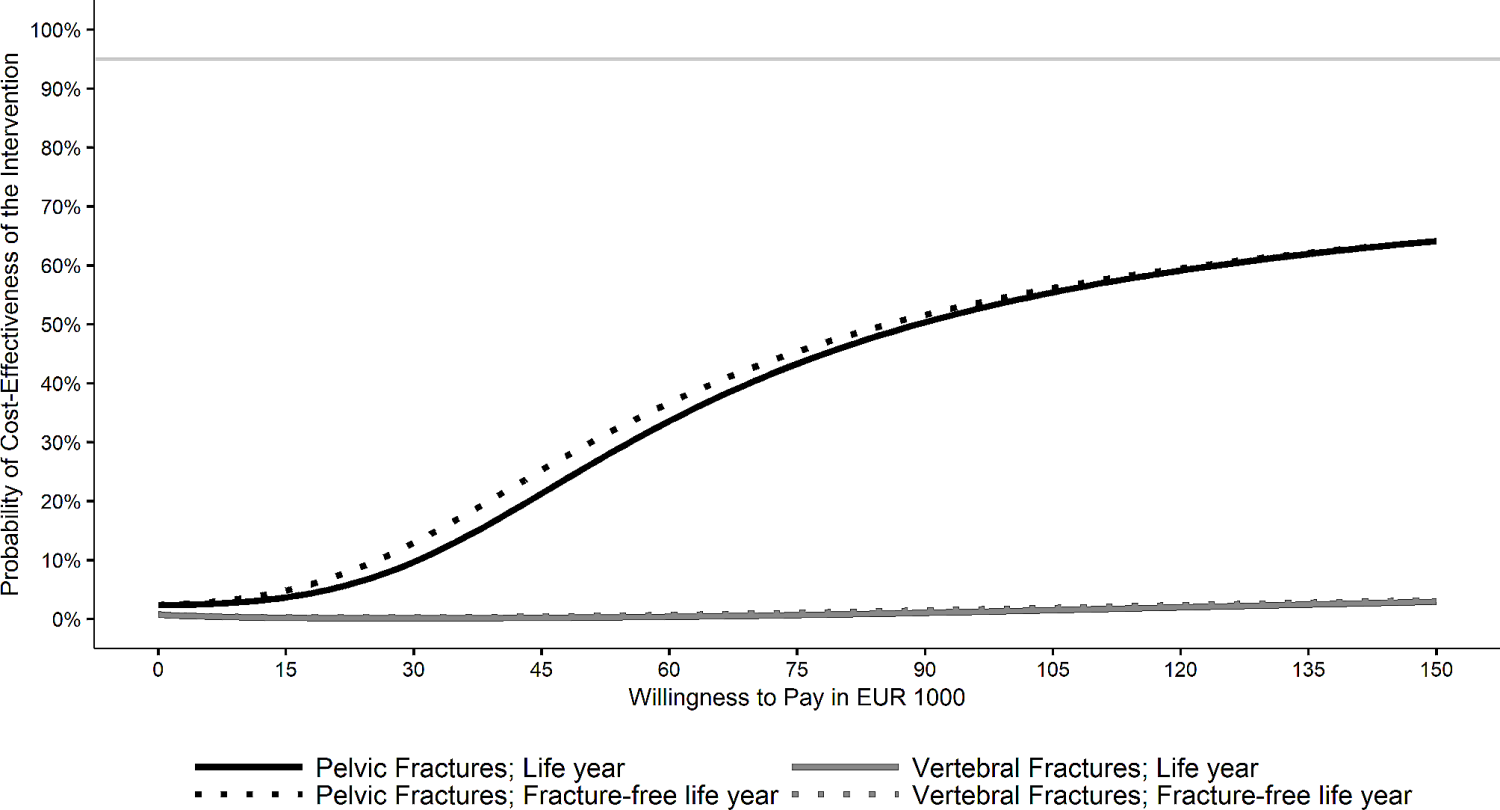
Cost-effectiveness acceptability curves for total costs per (fracture-free) life year gained

Results for the pelvic fracture cohort are displayed in Table 2. We obtained significantly higher total (€698) and inpatient (€538) costs in the OGCM group than in the non-OGCM group. The total length of stay in the OGCM group was significantly longer than in the non-OGCM group due to a significantly longer in-hospital stay. The length of stay in a rehabilitation facility, however, was significantly shorter in the OGCM than in the non-OGCM group. There were no differences concerning life years or fracture-free life years. The ICER was 89,473 per life year and 86,159 per fracture-free life year gained. The CEACs in Fig. 1 showed that the probability for treatment in an OGCM hospital to be cost-effective did not exceed 95% for a willingness-to-pay of up to €150,000 for neither effectiveness measure.

**Table 2:** Costs and outcome estimates for pelvic fractures

Outcome	OGCM group (*n* = 14,973)	Non-OGCM group (*n* = 6,063)	Difference	SE
Costs [€]				
Total^a^	22,572	21,875	698*	352
Inpatient^a^	11,288	10,750	538*	247
Thereof during index stay^a^	6,012	5,796	216	164
Medication^b^	1,391	1,381	10.03	44.87
Outpatient^b^	1,010	1,012	-2.11	24.24
Outpatient hospital^b^	22.87	19.87	3.01	2.81
Medical devices^b^	292	307	-15.36	10.73
Long-term care^c^	8,256	8,138	118	188
Length of stay [days]				
Total stay^a^	18.93	15.9	3.02***	0.4167
Thereof in hospital^a^	15.25	11.03	4.22***	0.2312
Thereof in rehabilitation facility^b^	3.68	4.88	-1.2***	0.2675
Effectiveness				
Life year^c^	0.8149	0.8071	0.0078	0.0094
Fracture-free life year^c^	0.7666	0.7584	0.0081	0.01
ICER				
€ per life year gained	89,473			
€ per fracture-free life year gained	86,159			

* *p* < .05; ** *p* < .01; *** *p* < .001

^a^ estimated with a gamma regression; ^b^ estimated with a two-part model with logistic and gamma part; ^c^ tested with a two sample t-test; OGCM = Orthogeriatric co-management; SE = Robust standard error

Results for the vertebral fracture cohort are displayed in Table 3. We obtained significantly higher total (€609), inpatient (€801), and index stay (€645) costs in the OGCM group than the non-OGCM group. Moreover, outpatient and medical devices costs were significantly lower after treatment in an OGCM compared to a non-OGCM hospital. The total and in-hospital length of stay in the OGCM group were significantly longer than in the non-OGCM group although the length of stay in a rehabilitation facility was significantly shorter in the OGCM than in the non-OGCM group. There was no significant difference regarding both effectiveness outcomes. Both ICERs showed that the OGCM group was dominated by the non-OGCM group.

**Table 3:** Costs and outcome estimates for vertebral fractures

Outcome	OGCM group (*n* = 24,633)	Non-OGCM group (*n* = 9,194)	Difference	SE
Costs [€]				
Total^a^	23,060	22,451	609*	251
Inpatient^a^	12,898	12,096	801***	187
Thereof during index stay^a^	6,675	6,030	645***	110
Medication^b^	1,458	1,449	9.38	29.18
Outpatient^b^	1,003	1,031	-27.99*	13.43
Outpatient hospital^b^	22.56	23.75	-1.2	1.84
Medical devices^b^	265	282	-16.12*	8.13
Long-term care^c^	7,089	7,165	-75.48	115
Length of stay [days]				
Total stay^a^	17.47	15.49	1.98***	0.306
Thereof in hospital^a^	14.92	11.57	3.35***	0.2258
Thereof in rehabilitation facility^b^	2.55	3.92	-1.37***	0.1617
Effectiveness				
Life year^c^	0.8363	0.8425	-0.0062	0.0056
Fracture-free life year^c^	0.7843	0.7916	-0.0073	0.006
ICER				
€ per life year gained	Dominated^d^			
€ per fracture-free life year gained	Dominated^d^			

* *p* < .05; ** *p* < .01; *** *p* < .001

^a^ estimated with a gamma regression; ^b^ estimated with a two-part model with logistic and gamma part; ^c^ tested with a two sample t-test; ^d^ OGCM was more costly and less effective than non-OGCM group; OGCM = Orthogeriatric co-management; SE = Robust standard error

Accounting for hospital clusters led to mostly similar results as the main analyses (supplementary Tables 3–4, supplementary Fig. 3). However, in the pelvic fracture cohort, the total (€1277) and inpatient cost differences (€937) between both groups were more pronounced and the index costs in the OGCM group were significantly higher (€844). There was no significant difference in length of stay in a rehabilitation facility here. The estimated ICERs were 232,265 per life year and €304,157 per fracture-free life year gained. Notable differences in the vertebral fracture cohort were significantly lower life years in the OGCM than in the non-OGCM group and no significant difference regarding outpatient costs. Similarly, the sensitivity analysis without balancing for hospital volume (supplementary Tables 5–6, supplementary Fig. 4), showed significantly higher total (€1370), inpatient (€1272), and index costs (€1071) in the OGCM group of the pelvic fracture cohort. Medication and outpatient hospital costs in the OGCM group were significantly higher, medical devices costs lower. In the vertebral fracture cohort, results were similar to the main analysis although total, inpatient, and index cost differences were more pronounced. Moreover, there was no significant difference between both groups in medical devices costs but significantly lower life years in the OGCM group.

## Discussion

In this retrospective cohort study with insurance claims data, we investigated geriatric patients with pelvic or vertebral fractures treated in hospitals that provided OGCM compared to hospitals that did not in a 1-year follow-up. Total costs were significantly higher in the OGCM than in the non-OGCM group for both fracture cohorts. We found no differences concerning life years or fracture-free life years in both cohorts. For both outcomes and cohorts, the probability for treatment in an OGCM hospital to be cost-effective did not exceed 95% for a willingness-to-pay of up to € 150,000.

Higher total and inpatient costs in the OGCM than the non-OGCM group stand in line with increased costs in the OGCM group of a similar German claims data study on hip fractures [45]. However, they are in contrast to both systematic reviews on orthogeriatric care for hip fractures [42, 43]. In the current study, like Schulz, Büchele [45], we found higher index stay costs in OGCM hospitals (albeit significantly higher in the pelvic fracture cohort only in the sensitivity analyses) and the total cost difference was mostly driven by these. In contrast, almost all studies from the systematic reviews reported lower index stay costs for orthogeriatric care [42, 43]. A longer length of stay [45] compared to mostly shorter inpatient stays in the other studies [42] might explain these divergences. Supporting the role of length of stay for the index cost difference, a single-center prospective cohort study [47] and a single-center retrospective cohort study [46] from Germany on inter alia pelvic and vertebral fractures did not find differences in length of stay [46, 47] or in costs between the OGCM and non-OGCM group [47]. Overall, comparing length of stay across different implementations of orthogeriatric care, countries, fracture locations, and study designs is difficult and likely rather relates to differences in health systems and structures than the quality of care [36, 48]. A distinct feature in Germany is the reimbursement of the OPS8-550 which might encourage hospitals to prolong the index stay at least to the 14-day threshold which triggers a higher reimbursement rate [51]. Moreover, a driving factor of the longer treatment duration in Germany might be the availability of complex occupational therapy and physiotherapy [48] and that patients received rehabilitative treatment during the index stay [34] instead of in separate rehabilitation facilities. Accordingly, we obtained a prolonged index hospital stay in OGCM hospitals but a longer stay in a subsequent rehabilitation facility in non-OGCM hospitals (in all but one sensitivity analysis).

For the other cost sectors, we only obtained small differences between both groups and none was significant across sensitivity analyses. In contrast to the investigation on hip fractures from Schulz, Büchele [45], we obtained no significant difference in long-term care costs between both groups although one study found institutionalization rates to be similar for vertebral, pelvic, and hip fractures [21]. However, Schulz, Büchele [45] found higher long-term care costs in the OGCM group only from a societal, not from a payer perspective while we only applied the latter.

Not finding benefits regarding life years in the OGCM groups stands in contrast to lower mortality rates or more life years in many studies on orthogeriatric care for hip fractures [37, 42, 43]. However, our results are in line with studies that included non-hip fractures and did not find reduced mortality for OGCM compared with a control group [48, 49], albeit one found a slight reduction [47]. Moreover, mortality rates in our cohorts were 27.7% (pelvic) and 24.8% (vertebral), which is comparable to 27.4% from Wiedl, Förch [70] who also used a 1-year follow-up and investigated inter alia similar fracture types. Possibly, small effects on mortality diminished over the follow-up period, considering that mortality after fragility fractures is highest shortly after the fracture event [71]. Moreover, there was no difference between both groups concerning fracture-free life years. However, re-fracture risk was shown to be highest immediately after the initial fracture [72] and to distinguish re-fracture and re-treatment we had to exclude all subsequent fracture diagnoses of the same fracture type in the first 6 weeks after the initial fracture.

In neither fracture cohort, the probability of treatment in an OGCM hospital being cost-effective exceeded 95% for a reasonable willingness-to-pay for neither of the effectiveness outcomes. In the vertebral fracture cohort, treatment in an OGCM hospital was dominated by the non-OGCM group. In contrast, treatment of hip fractures in an OGCM hospital was cost-effective at least at a substantial willingness-to-pay [45]. However, unlike vertebral and especially pelvic fractures, hip fractures usually demand an early surgical treatment [73] and the geriatrician’s role differs in OGCM when there is no indication for surgery [74]. Hence, it is still possible that treatment in an OGCM hospital might be cost-effective for patients with surgical treatment of pelvic or vertebral fractures or specific types of these fractures. Investigating this was beyond the scope of the current analysis and could be addressed in future studies.

### Limitations

A limitation of our study is that we had to assign cases to OGCM and non-OGCM group on hospital level. Hence, not all cases in the OGCM groups actually received OGCM. Accordingly, OPS8-550 was reimbursed in the OGCM groups for only 33.1% of the pelvic and 24.1% of vertebral fracture cases. Moreover, as we only considered the first treating hospital for group assignment, a few patients in the non-OGCM group might have received OGCM treatment after transference to a different hospital (OPS8-550 was reimbursed for ≤ 0.1% of the cases). While this might have led to an underestimation of the effectiveness of OGCM, patients who did not receive the treatment according to OPS8-550 probably still benefitted from the structures that hospitals established to be able to offer OGCM. In addition, we did not differentiate between conservative or surgical treatment and in our sample more than 30% of patients admitted with a vertebral fracture received an operative treatment which is not supported by evidence [75] and higher than in many other high-income countries. The high rate of operative treatments for vertebral fractures might be attributed to the German reimbursement scheme – payment for operative treatments is higher than for conservative treatments [76]. This might have affected the cost-effectiveness of treatment in OGCM-hospitals considering higher rates of operative treatments in the OGCM than the non-OGCM group.

Moreover, we excluded all cases treated in hospitals that frequently transferred patients to other care systems to ensure that cases were treated in a hospital of the group they were assigned to. This led to the exclusion of about 30,000 cases, limiting our scope to hospitals that rarely transfer to different care systems. Balancing for hospital volume could only be achieved with high weights for cases in the few hospitals in the control group with a high volume. Significantly higher index costs in the OGCM group of the pelvic fracture cohort in the analysis without balancing for hospital volume indicate that there likely was no significant difference in the main analysis due to cases in a few large but expensive non-OGCM hospitals that were assigned EB high weights. Overall, while higher differences in the index, inpatient, and total costs in both sensitivity analyses compared the main analysis, demand caution in the interpretation of the exact amount, they show that total, inpatient, and index costs (for vertebral fractures) differences as well as lacking benefits of OGCM hospitals are robust across scenarios.

Furthermore, not all relevant covariates can be found in insurance claims data. For example, we lacked information on the fractures’ severity, which might be a relevant confounder. Nevertheless, we used EB with a multitude of potentially confounding variables. Moreover, estimated costs only reflect the payer’s perspective and lack a societal view. A systematic review, however, found that the direct costs of fragility fractures outweigh the indirect or social costs [77]. Therefore, the payer perspective likely represents the majority of the relevant costs, especially considering the comprehensive reimbursement of health services by German statutory health insurances.

Lastly, we could not measure effectiveness with a generic outcome such as quality-adjusted life years [78]. Considering that OGCM for pelvic and vertebral fractures was positively associated with outcomes related to quality of life such as increased post-operative mobility [46, 47], it is possible that incorporating quality of life might lead to more favorable results. We used fracture-free life years as an additional effectiveness measure to take into account the increased risk for subsequent fragility fractures after an initial fracture [58]. However, incorporating survival and fracture-free time, interpretation of this outcome and comparison to other studies is difficult.

### Strengths

We used a large dataset of more than 50,000 cases of fragility fractures that allowed the incorporation of a multitude of covariates. Thus, we could balance the study groups for a range of baseline characteristics using EB. We used data from a health insurance association with a coverage of about one-third of the German population, which indicates that our results might be representative of the German population despite slight differences in population characteristics between insurance companies [79]. Moreover, some biases that might mitigate the validity of clinical research [80] might be less prominent in insurance claims data (e.g., there is no recall bias to be expected here). Lastly, this is, to our knowledge, the first study on the cost-effectiveness of OGCM for non-hip fractures (except for a recent study on forearm and humerus fractures by this research group [81]). While there is already little research on the effectiveness of OGCM for non-hip fractures, there is even less on its cost-effectiveness. Moreover, the studies that investigated OGCM for non-hip fractures mostly relied on in-hospital outcomes [46–50] while we applied a 1-year follow-up.

## Conclusion

In this analysis of claims data, we did not find treatment in an OGCM hospital to be cost-effective for a willingness-to-pay of up to €150,000. Yet, assigning cases to OGCM and non-OGCM group on hospital level might have led to an underestimation of OGCM’s benefits. Future studies could disentangle the impact of OGCM on health and economic outcomes for different fragility fracture locations and treatment options.

### Electronic supplementary material

Below is the link to the electronic supplementary material.

Supplementary Material 1: The file contains additional figures (a display of the hospital volume for both fracture cohorts and a flow-chart). Moreover, complete descriptive information are displayed here. In addition, all results and short descriptions of the sensitivity analyses are displayed within this file

## Data Availability

The datasets supporting the conclusions of this article are owned by the German statutory health insurance AOK. Since public deposition of the data would breach ethical and legal compliance, data are only available upon formal request from the research institute of the AOK (WIdO). To request the data please contact the institutional body of the WIdO (wido@wido.bv.aok.de). To fulfill the legal requirements to obtain that kind of data, researchers must obtain permission for a specific research question from the German Federal (Social) Insurance Office. Additionally, researchers must conclude a contract with the statutory health insurance regarding data access which can be requested from the “AOK-Bundesverband GbR” (Federal Association of Local Health Insurance Funds) under http://aok-bv.De/kontakt/. The licensee is permitted to use the data for the purpose of the research proposal within their company, exclusively. Thereby, a company is defined as an economic unit. Licensees are not allowed to pass the data to a third party, or to create software or databases except for scientific publications. Moreover, the study has to be approved by the data protection officer both at the statutory health insurance and the research institute.

## Abbreviations

CEAC: Cost-effectiveness acceptability curve
EB: Entropy balancing
ICER: Incremental cost-effectiveness ratio
OGCM: Orthogeriatric co-management
