# Supplementary material for: Health-economic evaluation of orthogeriatric co-management for patients with pelvic or vertebral fragility fractures
Source: BMC Geriatr. 2024 Aug 5;24:657. doi: 10.1186/s12877-024-05225-5 (PMC11302317; doi:10.1186/s12877-024-05225-5)

**Health-economic evaluation of orthogeriatric co-management for patients with pelvic or vertebral fragility fractures**

**Supplementary material**

**Supplementary Fig. 1** Flow-chart of study population

Study population

(n_p_ = 21,036; n_v_ = 33,827)

**Exclusions:**

Younger than 80 years (n_p_ = 8,293; n_v_ = 22,168)

Treated in hospital with > 5% change in care system (n_p_ = 12,071; n_v_ = 20,736)

Treated in hospital with very high number of cases (n_p_ = 292; n_v_ = 579)

Implausibly low index charges (n_p_ = 0; n_v_ = 1)

Insufficient insurance status for baseline or follow-up (n_p_ = 22; n_v_ = 57)

Fracture in washout-period (n_p_ = 175; n_v_ = 477)

Inpatient hospital stay after death (n_p_ = 9; n_v_ = 18)

Patients treated in OGCM hospitals

(n = 24,633)

Patients treated in non-OGCM hospitals

(n = 9,194)

Vertebral fracture cohort

(n = 33,827)

With inpatient stay due to fragility fracture in 2014-2018

(n_p_ = 41,898; n_v_ = 77,863)

Pelvic fracture cohort

(n = 21,036)

Patients treated in OGCM hospitals

(n = 14,973)

Patients treated in non-OGCM hospitals

(n = 6,063)

n_p_: Number of patients with pelvic fractures; n_v_: Number of patients with vertebral fractures

**Supplementary Fig. 2** Hospital volume (fracture cases per hospital) for OGCM and non-OGCM hospitals in both cohorts


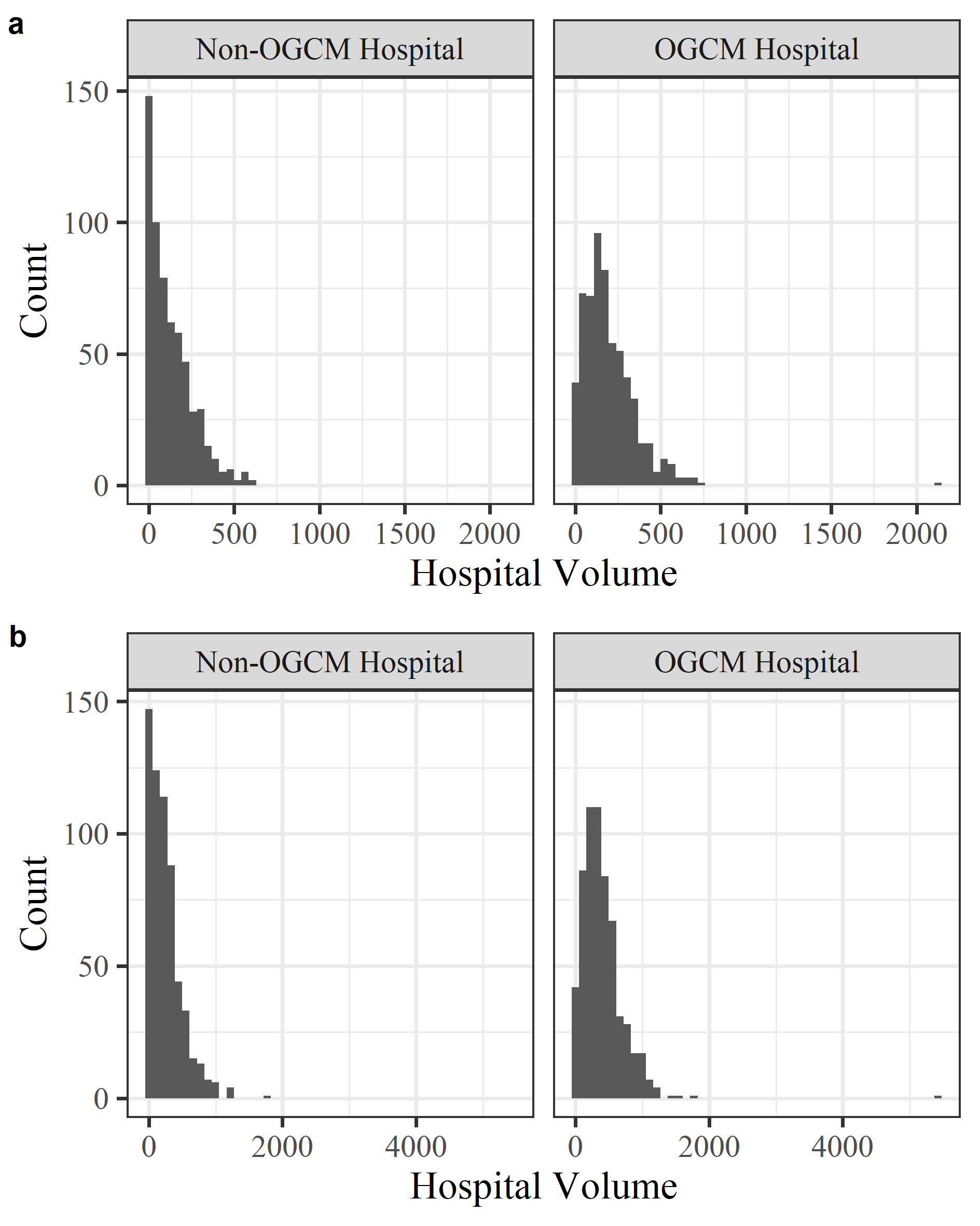


These values were weighted by the insurer’s coverage in the respective federal state. a: Pelvic fracture cohort, b: Vertebral fracture cohort. All cases belonging to the outlier in the OGCM hospital group were excluded during data preprocessing.

| **Supplementary table 1** Medication based comorbidities before and after EB for pelvic fractures | | | |
| --- | --- | --- | --- |
| Baseline: 1 year | OGCM group (N=14,973) | Non-OGCM group (N=6,063) | |
|  |  | Before EB | After EB |
| Medication-based comorbidities [%] | | | |
| Acid related disorders | 52.09 (49.96) | 52.07 (49.96) | 52.09 (49.96) |
| Bone diseases (osteoporosis) | 12.22 (32.75) | 13 (33.63) | 12.21 (32.74) |
| Cancer | 0.64 (7.98) | 0.58 (7.58) | 0.64 (8) |
| Cardiovascular diseases (incl.   hypertension) | 88.78 (31.56) | 88.37 (32.06) | 88.78 (31.56) |
| Dementia | 7.59 (26.48) | 7.03 (25.56) | 7.59 (26.48) |
| Diabetes mellitus | 19.47 (39.6) | 20.47 (40.35) | 19.47 (39.6) |
| Epilepsy | 10 (30) | 9.93 (29.91) | 10 (30) |
| Glaucoma | 9.7 (29.59) | 10.09 (30.13) | 9.69 (29.58) |
| Gout, Hyperuricemia | 11.87 (32.34) | 13 (33.63) | 11.87 (32.34) |
| HIV | Excluded due to less than 50 observations | | |
| Hyperlipidemia | 30.21 (45.92) | 30.23 (45.93) | 30.22 (45.92) |
| Intestinal inflammatory diseases | 0.91 (9.52) | 1.14 (10.61) | 0.91 (9.52) |
| Iron deficiency anemia | 7.75 (26.75) | 6.78 (25.14) | 7.75 (26.74) |
| Migraines | Excluded due to less than 50 observations | | |
| Pain | 64.1 (47.97) | 61.26 (48.72) | 64.1 (47.97) |
| Parkinson’s disease | 6.93 (25.4) | 6.61 (24.85) | 6.93 (25.4) |
| Psychological disorders (sleep disorder,   depression) | 35.58 (47.88) | 37.09 (48.31) | 35.59 (47.88) |
| Psychoses | 17.06 (37.62) | 17.05 (37.61) | 17.07 (37.63) |
| Respiratory illness (asthma, COPD) | 13.64 (34.33) | 13.69 (34.38) | 13.64 (34.32) |
| Rheumatologic conditions | 33.69 (47.27) | 35.31 (47.8) | 33.68 (47.26) |
| Thyroid disorders | 23.74 (42.55) | 24.08 (42.76) | 23.75 (42.56) |
| Tuberculosis | Excluded due to less than 50 observations | | |
| Standard deviation is stated in parentheses; EB = Entropy balancing; OGCM = Orthogeriatric co-management | | | |

| **Supplementary table 2** Descriptive statistics before and after EB for vertebral fractures | | | |
| --- | --- | --- | --- |
| Baseline: 1 year | OGCM group (N=24,633) | Non-OGCM group (N=9,194) | |
|  |  | Before EB | After EB |
| Female gender [%] | 76.07 (42.66) | 76.59 (42.34) | 76.09 (42.65) |
| Age: Mean [years] | 86.21 (4.26) | 86.11 (4.17) | 86.21 (4.26) |
| Vertebral fracture cases per hospital: Mean | 614 (315) | 449 (257) | 612 (316) |
| Treatment in 2014 [%] | 16.7 (37.3) | 23.34 (42.3) | 16.73 (37.32) |
| Treatment in 2015 [%] | 18.58 (38.9) | 20.6 (40.45) | 18.58 (38.89) |
| Treatment in 2016 [%] | 20.16 (40.12) | 20.09 (40.07) | 20.17 (40.13) |
| Treatment in 2017 [%] | 21.9 (41.36) | 17.53 (38.03) | 21.89 (41.35) |
| Care dependence during baseline: Mean [months] | | | |
| nursing home | 1.27 (3.5) | 1.26 (3.47) | 1.27 (3.5) |
| care level 1 | 0.06 (0.63) | 0.06 (0.67) | 0.06 (0.63) |
| care level 2 | 2.87 (4.72) | 2.69 (4.61) | 2.87 (4.72) |
| care level 3 | 1.72 (3.85) | 1.69 (3.82) | 1.72 (3.85) |
| care level 4 | 0.86 (2.86) | 0.84 (2.82) | 0.86 (2.86) |
| care level 5 | 0.12 (1.09) | 0.1 (0.98) | 0.12 (1.09) |
| Costs during baseline: Mean [€] | | | |
| for inpatient hospital treatment | 4,377 (7,257) | 3,880 (6,582) | 4,376 (7,254) |
| for medication | 1,302 (1,604) | 1,224 (1,505) | 1,302 (1,604) |
| for outpatient treatment | 1,128 (911) | 1,145 (871) | 1,129 (912) |
| for outpatient hospital treatment | 24.15 (112) | 22.07 (100) | 24.18 (112) |
| for medical devices | 178 (409) | 171 (390) | 178 (409) |
| for long-term care | 4,976 (6,078) | 4,805 (6,023) | 4,975 (6,077) |
| Medication-based comorbidities [%] | | | |
| Acid related disorders | 53.82 (49.85) | 53.8 (49.86) | 53.83 (49.85) |
| Bone diseases (osteoporosis) | 13.68 (34.37) | 14.06 (34.77) | 13.68 (34.37) |
| Cancer | 0.68 (8.21) | 0.62 (7.85) | 0.68 (8.21) |
| Cardiovascular diseases (incl.   hypertension) | 88.27 (32.18) | 87.81 (32.72) | 88.28 (32.17) |
| Dementia | 6.34 (24.36) | 6.32 (24.33) | 6.34 (24.36) |
| Diabetes mellitus | 20.51 (40.38) | 20.38 (40.29) | 20.5 (40.37) |
| Epilepsy | 11.05 (31.36) | 10.8 (31.04) | 11.05 (31.35) |
| Glaucoma | 9.63 (29.5) | 9.63 (29.5) | 9.62 (29.49) |
| Gout, Hyperuricemia | 12.45 (33.01) | 13.32 (33.99) | 12.45 (33.01) |
| HIV | Excluded due to less than 50 observations | | |
| Hyperlipidemia | 33.13 (47.07) | 31.92 (46.62) | 33.13 (47.07) |
| Intestinal inflammatory diseases | 1.08 (10.32) | 1.11 (10.47) | 1.08 (10.31) |
| Iron deficiency anemia | 6.52 (24.69) | 6.07 (23.88) | 6.52 (24.69) |
| Migraines | Excluded due to less than 50 observations | | |
| Pain | 67.88 (46.69) | 66.06 (47.35) | 67.89 (46.69) |
| Parkinson’s disease | 7.74 (26.73) | 7.85 (26.9) | 7.75 (26.73) |
| Psychological disorders (sleep disorder,   depression) | 33.67 (47.26) | 35.36 (47.81) | 33.67 (47.26) |
| Psychoses | 13.78 (34.47) | 14.36 (35.07) | 13.77 (34.46) |
| Respiratory illness (asthma, COPD) | 15.92 (36.58) | 14.74 (35.45) | 15.91 (36.58) |
| Rheumatologic conditions | 38.69 (48.7) | 41.08 (49.2) | 38.7 (48.71) |
| Thyroid disorders | 23.85 (42.62) | 24.74 (43.16) | 23.84 (42.61) |
| Tuberculosis | Excluded due to less than 50 observations | | |
| Standard deviation is stated in parentheses; EB = Entropy balancing; OGCM = Orthogeriatric co-management | | | |

**Sensitivity Analysis: Accounting for clusters**

To account for clusters introduced by cases being treated in the same hospitals, we recalculated the analyses including random intercepts for hospitals. However, we could not recalculate all models in this manner as random intercepts included in both, the logistic regression of the first part and the generalized linear regression with gamma distribution of the second part of two-part models, led to non-convergence. Thus, we only applied a random intercept in the logistic regression (first part) as in this way the random intercept term did not only affect cases for which costs occurred. The reported incremental cost-effectiveness ratios reflect the relations of the differences between OGCM and non-OGCM groups estimated by the respective model of total costs and effectiveness. Lastly, we report cost-effectiveness acceptability curves based on net-monetary regressions that also included random intercepts for hospitals. Estimated costs and outcomes can be found in supplementary Tables 3 and 4 and the cost-effectiveness acceptability curves can be found in supplementary Fig 3.

| **Supplementary table 3** Costs and outcome estimates for pelvic fractures estimated with random intercept for hospitals | | | | |
| --- | --- | --- | --- | --- |
| Outcome | OGCM group  (n = 14,973) | Non-OGCM group  (n = 6,063) | Difference | SE |
| Costs [€] |  |  |  |  |
| Total^a^ | 22,529 | 21,252 | 1,277*** | 319 |
| Inpatient^a^ | 11,109 | 10,172 | 937*** | 281 |
| Thereof during index stay^a^ | 5,817 | 4,974 | 844*** | 234 |
| Medication^b^ | 1,399 | 1,391 | 7.51 | 50.17 |
| Outpatient^b^ | 1,016 | 1,023 | -7.07 | 31.67 |
| Outpatient hospital^b^ | 17.28 | 16.01 | 1.27 | 3.59 |
| Medical devices^b^ | 292 | 299 | -7.11 | 11.84 |
| Long-term care^c^ | 8,257 | 8,080 | 177 | 136 |
| Length of stay [days] |  |  |  |  |
| Total stay^a^ | 18.43 | 14.91 | 3.52*** | 0.5612 |
| Thereof in hospital^a^ | 15.09 | 11.05 | 4.04*** | 0.4002 |
| Thereof in rehabilitation facility^b^ | 1.81 | 2.35 | -0.5407 | 0.2761 |
| Effectiveness |  |  |  |  |
| Life year^c^ | 0.8176 | 0.8121 | 0.0055 | 0.0066 |
| Fracture-free life year^c^ | 0.7685 | 0.7643 | 0.0042 | 0.0071 |
| ICER |  |  |  |  |
| € per life year gained | 232,265 |  |  |  |
| € per fracture-free life year gained | 304,157 |  |  |  |
| * *p* < .05; ** *p* < .01; *** *p* < .001 ^a^ estimated with a gamma regression with random intercept term for hospitals; ^b^ estimated with a two-part model with logistic and gamma part with random intercept term for hospitals in logistic part; ^c^ estimated with a linear mixed regression with random intercept term for hospitals; OGCM = Orthogeriatric co-management; SE = Robust standard error | | | | |

| **Supplementary table 4** Costs and outcome estimates for vertebral fractures estimated with random intercept for hospitals | | | | |
| --- | --- | --- | --- | --- |
| Outcome | OGCM group  (n = 24,633) | Non-OGCM group  (n = 9,194) | Difference | SE |
| Costs [€] |  |  |  |  |
| Total^a^ | 22,917 | 22,267 | 650* | 267 |
| Inpatient^a^ | 12,600 | 11,894 | 706** | 239 |
| Thereof during index stay^a^ | 6,277 | 5,568 | 710*** | 155 |
| Medication^b^ | 1,464 | 1,451 | 12.79 | 28.78 |
| Outpatient^b^ | 1,007 | 1,038 | -31.28 | 33.34 |
| Outpatient hospital^b^ | 17.63 | 17.51 | 0.121 | 1.65 |
| Medical devices^b^ | 265 | 283 | -18.37* | 8.74 |
| Long-term care^c^ | 7,087 | 7,040 | 47.03 | 112 |
| Length of stay [days] |  |  |  |  |
| Total stay^a^ | 16.78 | 14.42 | 2.35*** | 0.4446 |
| Thereof in hospital^a^ | 14.36 | 11.54 | 2.83*** | 0.3819 |
| Thereof in rehabilitation facility^b^ | 1.39 | 2.03 | -0.638*** | 0.1655 |
| Effectiveness |  |  |  |  |
| Life year^c^ | 0.8371 | 0.8483 | -0.0112* | 0.0049 |
| Fracture-free life year^c^ | 0.7854 | 0.7952 | -0.0098 | 0.0056 |
| ICER |  |  |  |  |
| € per life year gained | Dominated^d^ |  |  |  |
| € per fracture-free life year gained | Dominated^d^ |  |  |  |
| * *p* < .05; ** *p* < .01; *** *p* < .001 ^a^ estimated with a gamma regression with random intercept term for hospitals; ^b^ estimated with a two-part model with logistic and gamma part with random intercept term for hospitals in logistic part; ^c^ estimated with a linear mixed regression with random intercept term for hospitals; ^d^ OGCM was more costly and less effective than non-OGCM group; OGCM = Orthogeriatric co-management; SE = Robust standard error | | | | |

**Supplementary Fig. 3** Cost-effectiveness acceptability curves for total costs estimated with random intercepts for hospitals

**
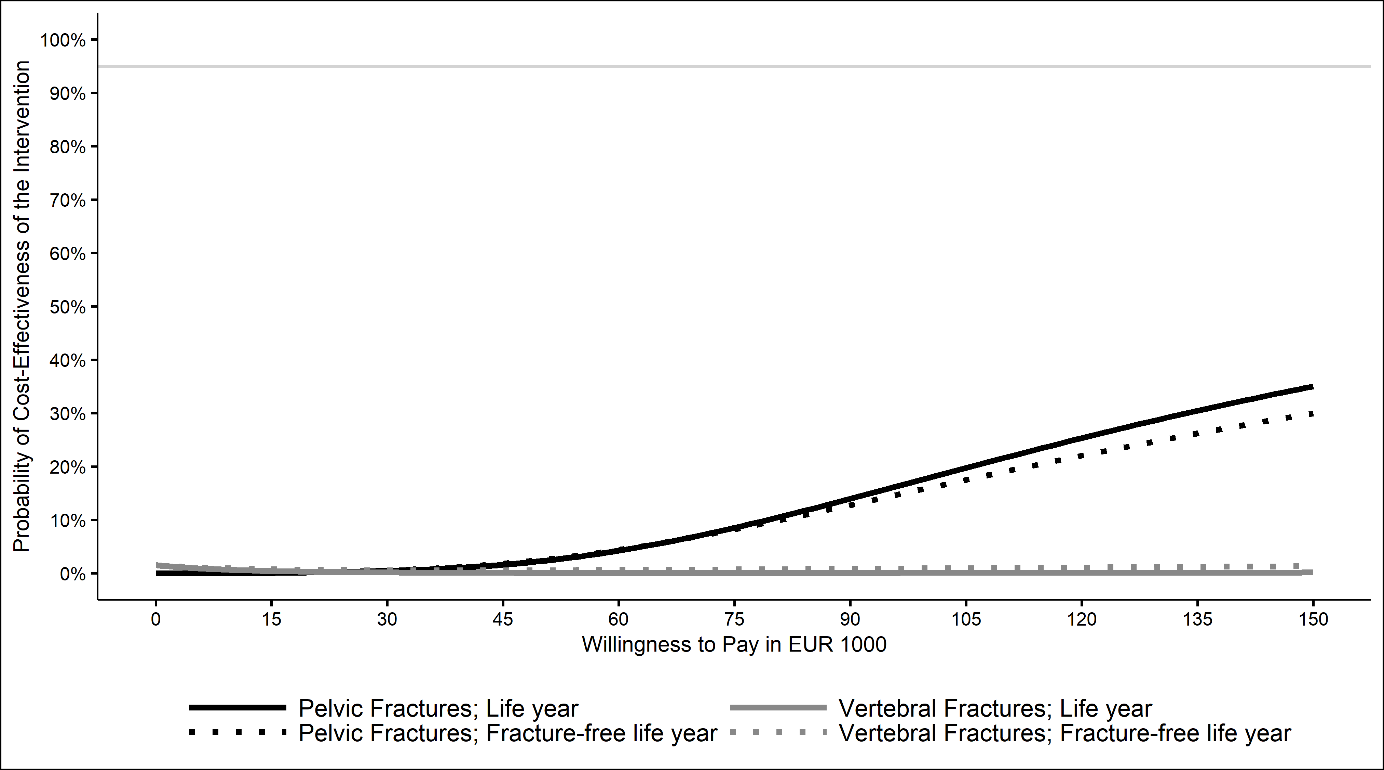
**

**Sensitivity analyses: Without balancing for hospital volume**

| **Supplementary table 5** Costs and outcome estimates for pelvic fractures estimated without balancing for hospital volume | | | | |
| --- | --- | --- | --- | --- |
| Outcome | OGCM group  (n = 14,973) | Non-OGCM group  (n = 6,063) | Difference | SE |
| Costs [€] |  |  |  |  |
| Total^a^ | 22,572 | 21,202 | 1370*** | 202 |
| Inpatient^a^ | 11,288 | 10,015 | 1272*** | 140 |
| Thereof during index stay^a^ | 6,012 | 4,941 | 1071*** | 61.5 |
| Medication^b^ | 1,391 | 1,341 | 50.4* | 23.15 |
| Outpatient^b^ | 1,010 | 1,039 | -29.05 | 16.28 |
| Outpatient hospital^b^ | 22.87 | 18.99 | 3.88* | 1.55 |
| Medical devices^b^ | 292 | 307 | -15.15* | 7.01 |
| Long-term care^c^ | 8,256 | 8,152 | 104 | 106 |
| Length of stay [days] |  |  |  |  |
| Total stay^a^ | 18.93 | 15.06 | 3.87*** | 0.2264 |
| Thereof in hospital^a^ | 15.25 | 10.58 | 4.67*** | 0.1483 |
| Thereof in rehabilitation facility^b^ | 3.68 | 4.47 | -0.7975*** | 0.1437 |
| Effectiveness |  |  |  |  |
| Life year^c^ | 0.8149 | 0.8141 | 0.0008 | 0.0053 |
| Fracture-free life year^c^ | 0.7666 | 0.7685 | -0.0019 | 0.0056 |
| ICER |  |  |  |  |
| € per life year gained | 1,712,781 |  |  |  |
| € per fracture-free life year gained | Dominated^d^ |  |  |  |
| * *p* < .05; ** *p* < .01; *** *p* < .001 ^a^ estimated with a gamma regression; ^b^ estimated with a two-part model with logistic and gamma part; ^c^ tested with a two sample t-test; OGCM = Orthogeriatric co-management; SE = Standard error; ^d^ OGCM was more costly and less effective than non-OGCM group; OGCM = Orthogeriatric co-management; SE = Robust standard error | | | | |

| **Supplementary table 6** Costs and outcome estimates for vertebral fractures estimated without balancing for hospital volume | | | | |
| --- | --- | --- | --- | --- |
| Outcome | OGCM group  (n = 24,633) | Non-OGCM group  (n = 9,194) | Difference | SE |
| Costs [€] |  |  |  |  |
| Total^a^ | 23,060 | 22,267 | 793*** | 184 |
| Inpatient^a^ | 12,898 | 11,924 | 974*** | 142 |
| Thereof during index stay^a^ | 6,675 | 5,685 | 989*** | 70.05 |
| Medication^b^ | 1,458 | 1,450 | 7.79 | 20.48 |
| Outpatient^b^ | 1,003 | 1,047 | -44.51*** | 10.25 |
| Outpatient hospital^b^ | 22.56 | 23.85 | -1.29 | 1.33 |
| Medical devices^b^ | 265 | 276 | -10.6 | 5.72 |
| Long-term care^c^ | 7,089 | 7,182 | -93 | 80.98 |
| Length of stay [days] |  |  |  |  |
| Total stay^a^ | 17.47 | 14.48 | 2.99*** | 0.202 |
| Thereof in hospital^a^ | 14.92 | 11.11 | 3.81*** | 0.159 |
| Thereof in rehabilitation facility^b^ | 2.55 | 3.38 | -0.8269*** | 0.1009 |
| Effectiveness |  |  |  |  |
| Life year^c^ | 0.8363 | 0.845 | -0.0087* | 0.004 |
| Fracture-free life year^c^ | 0.7843 | 0.7906 | -0.0062 | 0.0043 |
| ICER |  |  |  |  |
| € per life year gained | Dominated^d^ |  |  |  |
| € per fracture-free life year gained | Dominated^d^ |  |  |  |
| * *p* < .05; ** *p* < .01; *** *p* < .001 ^a^ estimated with a gamma regression; ^b^ estimated with a two-part model with logistic and gamma part; ^c^ tested with a two sample t-test; OGCM = Orthogeriatric co-management; SE = Standard error; ^d^ OGCM was more costly and less effective than non-OGCM group; OGCM = Orthogeriatric co-management; SE = Robust standard error | | | | |

**Supplementary Fig. 4** Cost-effectiveness acceptability curves for total costs estimated without balancing for hospital volume


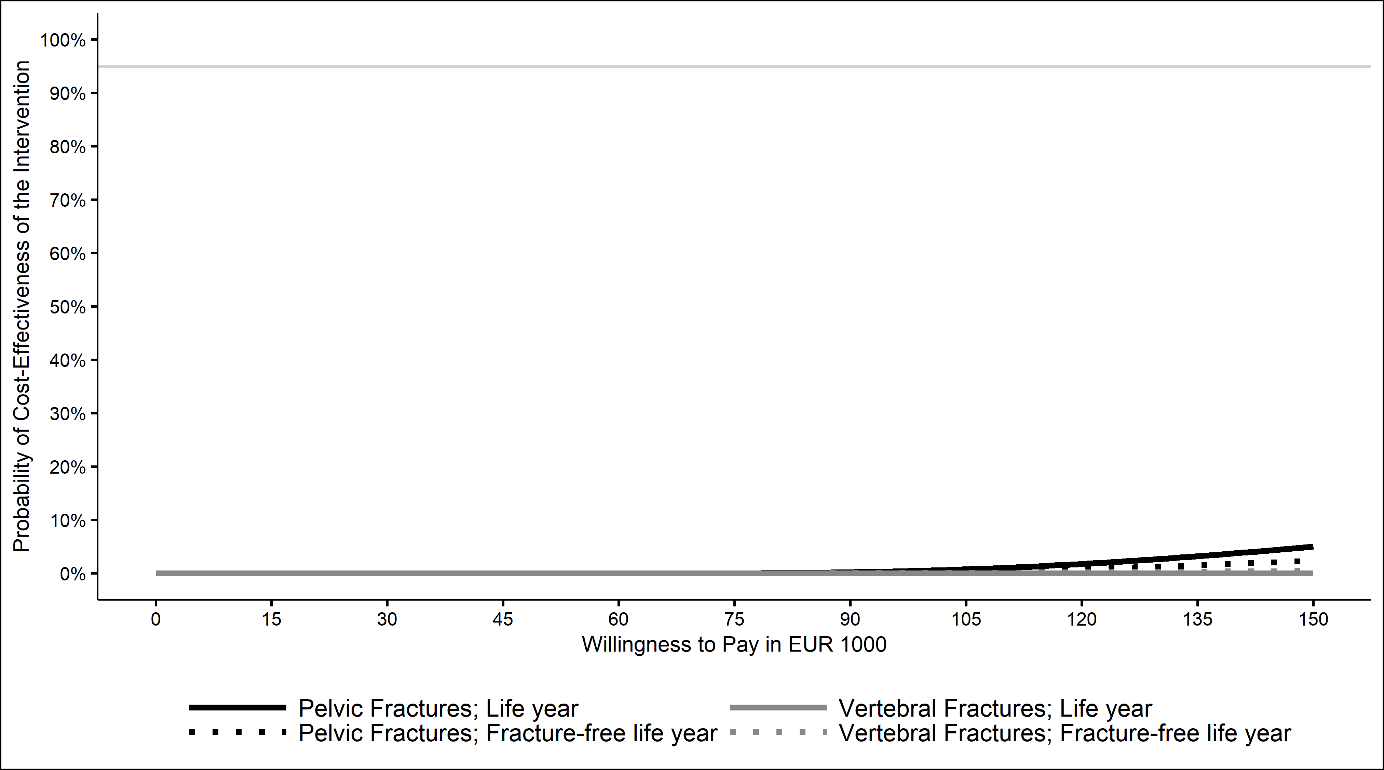

Supplement: Supplementary file 1 — Supplementary Material 1: The file contains additional figures (a display of the hospital volume for both fracture cohorts and a flow-chart). Moreover, complete descriptive information are displayed here. In addition, all results and short descriptions of the sensitivity analyses are displayed within this file [file 12877_2024_5225_MOESM1_ESM.docx]
